# Hyperglycemia at admission, comorbidities, and in-hospital mortality in elderly patients hospitalized in internal medicine wards: data from the RePoSI Registry

**DOI:** 10.1007/s00592-021-01716-8

**Published:** 2021-04-22

**Authors:** Salvatore Corrao, Alessandro Nobili, Giuseppe Natoli, Pier Mannuccio Mannucci, Francesco Perticone, Antonello Pietrangelo, Christiano Argano, on behalf of the REPOSI Investigators, Pier Mannuccio Mannucci, Alessandro Nobili, Antonello Pietrangelo, Francesco Perticone, Giuseppe Licata, Francesco Violi, Gino Roberto Corazza, Salvatore Corrao, Alessandra Marengoni, Francesco Salerno, Matteo Cesari, Mauro Tettamanti, Luca Pasina, Carlotta Franchi, Carlotta Franchi, Laura Cortesi, Mauro Tettamanti, Gabriella Miglio, Mauro Tettamanti, Laura Cortesi, Ilaria Ardoino, Alessio Novella, Domenico Prisco, Elena Silvestri, Giacomo Emmi, Alessandra Bettiol, Cenci Caterina, Gianni Biolo, Michela Zanetti, Martina Guadagni, Michele Zaccari, Massimiliano Chiuch, Michele Zaccari, Massimo Vanoli, Giulia Grignani, Edoardo Alessandro Pulixi, Mauro Bernardi, Silvia Li Bassi, Luca Santi, Giacomo Zaccherini, Graziana Lupattelli, Elmo Mannarino, Vanessa Bianconi, Francesco Paciullo, Riccardo Alcidi, Ranuccio Nuti, Roberto Valenti, Martina Ruvio, Silvia Cappelli, Alberto Palazzuoli, Domenico Girelli, Fabiana Busti, Giacomo Marchi, Mario Barbagallo, Ligia Dominguez, Floriana Cocita, Vincenza Beneduce, Lidia Plances, Giuseppe Natoli, Salvatore Mularo, Massimo Raspanti, Christiano Argano, Marco Zoli, Ilaria Lazzari, Mattia Brunori, Elisa Fabbri, Donatella Magalotti, Raffaella Arnò, Franco Laghi Pasini, Pier Leopoldo Capecchi, Giuseppe Palasciano, Maria Ester Modeo, Carla Di Gennaro, Maria Domenica Cappellini, Diletta Maira, Valeria Di Stefano, Giovanna Fabio, Sonia Seghezzi, Marta Mancarella, Margherita Migone De Amicis, Giacomo De Luca, Natalia Scaramellini, Matteo Cesari, Paolo Dionigi Rossi, Sarah Damanti, Marta Clerici, Federica Conti, Giulia Bonini, Barbara Brignolo Ottolini, Antonio Di Sabatino, Emanuela Miceli, Marco Vincenzo Lenti, Martina Pisati, Costanza Caccia Dominioni, Giovanni Murialdo, Alessio Marra, Federico Cattaneo, Roberto Pontremoli, Valentina Beccati, Giulia Nobili, Maria Beatrice Secchi, Davide Ghelfi, Luigi Anastasio, Lucia Sofia, Maria Carbone, Francesco Cipollone, Maria Teresa Guagnano, Emanuele Valeriani, Ilaria Rossi, Gerardo Mancuso, Daniela Calipari, Mosè Bartone, Giuseppe Delitala, Maria Berria, Chiara Pes, Alessandro Delitala, Maurizio Muscaritoli, Alessio Molfino, Enrico Petrillo, Giuseppe Zuccalà, Gabriella D’Aurizio, Giuseppe Romanelli, Alessandra Marengoni, Alberto Zucchelli, Francesca Manzoni, Andrea Volpini, Antonio Picardi, Umberto Vespasiani Gentilucci, Paolo Gallo, Chiara Dell’Unto, Giorgio Annoni, Maurizio Corsi, Giuseppe Bellelli, Sara Zazzetta, Paolo Mazzola, Hajnalka Szabo, Alessandra Bonfanti, Franco Arturi, Elena Succurro, Mariangela Rubino, Bruno Tassone, Giorgio Sesti, Maria Grazia Serra, Maria Antonietta Bleve, Laura Gasbarrone, Maria Rosaria Sajeva, Antonio Brucato, Silvia Ghidoni, Fabrizio Fabris, Irene Bertozzi, Giulia Bogoni, Maria Victoria Rabuini, Elisabetta Cosi, Paolo Scarinzi, Annalisa Amabile, Elisabetta Omenetto, Tancredi Prandini, Roberto Manfredini, Fabio Fabbian, Benedetta Boari, Alfredo De Giorgi, Ruana Tiseo, Roberto De Giorgio, Giuseppe Paolisso, Maria Rosaria Rizzo, Claudio Borghi, Enrico Strocchi, Eugenia Ianniello, Mario Soldati, Carlo Sabbà, Francesco Saverio Vella, Patrizia Suppressa, Andrea Schilardi, Francesca Loparco, Giovanni Michele De Vincenzo, Alessio Comitangelo, Emanuele Amoruso, Luigi Fenoglio, Andrea Falcetta, Christian Bracco, Anna L. Fracanzani Silvia Fargion, Silvia Tiraboschi, Annalisa Cespiati, Giovanna Oberti, Giordano Sigon, Flora Peyvandi, Raffaella Rossio, Barbara Ferrari, Giulia Colombo, Pasquale Agosti, Valter Monzani, Valeria Savojardo, Christian Folli, Giuliana Ceriani, Francesco Salerno, Giada Pallini, Franco Dallegri, Luciano Ottonello, Luca Liberale, Lara Caserza, Kassem Salam, Nicola Lucio Liberato, Tiziana Tognin, Giovanni Battista Bianchi, Sabrina Giaquinto, Francesco Purrello, Antonino Di Pino, Salvatore Piro, Renzo Rozzini, Lina Falanga, Elena Spazzini, Camillo Ferrandina, Giuseppe Montrucchio, Paolo Petitti, Paolo Peasso, Edoardo Favale, Cesare Poletto, Raffaella Salmi, Piergiorgio Gaudenzi, Francesco Violi, Ludovica Perri, Raffaele Landolfi, Massimo Montalto, Antonio Mirijello, Luigina Guasti, Luana Castiglioni, Andrea Maresca, Alessandro Squizzato, Leonardo Campiotti, Alessandra Grossi, Marco Bertolotti, Chiara Mussi, Giulia Lancellotti, Maria Vittoria Libbra, Giulia Dondi, Elisa Pellegrini, Lucia Carulli, Matteo Galassi, Yasmine Grassi, Maria Perticone, Rosa Battaglia, Marco FIlice, Raffaele Maio, Vincenzo Stanghellini, Eugenio Ruggeri, Sara del Vecchio, Andrea Salvi, Roberto Leonardi, Giampaolo Damiani, William Capeci, Armando Gabrielli, Massimo Mattioli, Giuseppe Pio Martino, Lorenzo Biondi, Pietro Pettinari, Riccardo Ghio, Anna Dal Col, Salvatore Minisola, Luciano Colangelo, Mirella Cilli, Giancarlo Labbadia, Antonella Afeltra, Benedetta Marigliano, Maria Elena Pipita, Pietro Castellino, Luca Zanoli, Samuele Pignataro, Alfio Gennaro, Julien Blanco, Valter Saracco, Marisa Fogliati, Carlo Bussolino, Francesca Mete, Miriam Gino, Antonio Cittadini, Carlo Vigorito, Michele Arcopinto, Andrea Salzano, Emanuele Bobbio, Alberto Maria Marra, Domenico Sirico, Guido Moreo, Francesca Gasparini, Silvia Prolo, Gloria Pina, Alberto Ballestrero, Fabio Ferrando, Sergio Berra, Simonetta Dassi, Maria Cristina Nava, Bruno Graziella, Stefano Baldassarre, Salvatore Fragapani, Gabriella Gruden, Giorgio Galanti, Gabriele Mascherini, Cristian Petri, Laura Stefani, Margherita Girino, Valeria Piccinelli, Francesco Nasso, Vincenza Gioffrè, Maria Pasquale, Giuseppe Scattolin, Sergio Martinelli, Mauro Turrin, Leonardo Sechi, Cristina Catena, Gianluca Colussi, Nicola Passariello, Luca Rinaldi, Franco Berti, Giuseppe Famularo, Patrizia Tarsitani, Roberto Castello, Michela Pasino, Gian Paolo Ceda, Marcello Giuseppe Maggio, Simonetta Morganti, Andrea Artoni, Stefano Del Giacco, Davide Firinu, Francesca Losa, Giovanni Paoletti, Giulia Costanzo, Giuseppe Montalto, Anna Licata, Valentina Malerba, Filippo Alessandro Montalto, Antonino Lasco, Giorgio Basile, Antonino Catalano, Lorenzo Malatino, Benedetta Stancanelli, Valentina Terranova, Salvatore Di Marca, Rosario Di Quattro, Lara La Malfa, Rossella Caruso, Patrizia Mecocci, Carmelinda Ruggiero, Virginia Boccardi, Tiziana Meschi, Fulvio Lauretani, Andrea Ticinesi, Antonio Nouvenne, Pietro Minuz, Luigi Fondrieschi, Mario Pirisi, Gian Paolo Fra, Daniele Sola, Massimo Porta, Piero Riva, Roberto Quadri, Erica Larovere, Marco Novelli, Giorgio Scanzi, Caterina Mengoli, Stella Provini, Laura Ricevuti, Emilio Simeone, Rosa Scurti, Fabio Tolloso, Roberto Tarquini, Alice Valoriani, Silvia Dolenti, Giulia Vannini, Alberto Tedeschi, Lucia Trotta, Riccardo Volpi, Pietro Bocchi, Alessandro Vignali, Sergio Harari, Chiara Lonati, Mara Cattaneo, Federico Napoli

**Affiliations:** 1 Department of Internal Medicine, UOC Medicina Interna 2 iGR, National Relevance Hospital Trust, ARNAS Civico, Di Cristina e Benfratelli, Piazza Nicola Leotta, 4 – 90127 Palermo, Italy; 2 Biomedical Department of Internal Medicine and Medical Specialties (DiBiMIS), University of Palermo, Palermo, Italy; 3 Department of Neuroscience, IRCCS Istituto Di Ricerche Farmacologiche Mario Negri, Milan, Italy; 4 Scientific Direction, IRCCS Foundation Maggiore Hospital Policlinico, Milan, Italy; 5 Department of Medical and Surgical Sciences, University Magna Graecia of Catanzaro, Catanzaro, Italy; 6 Department of Internal Medicine II, CenterforHemochromatosis, University of Modena and Reggio Emilia Policlinico, Modena, Italy

**Keywords:** Elderly, Hyperglycemia, Diabetes, Disability, Comorbidity, Mortality

## Abstract

**Aims:**

The association between hyperglycemia at hospital admission and relevant short- and long-term outcomes in elderly population is known. We assessed the effects on mortality of hyperglycemia, disability, and multimorbidity at admission in internal medicine ward in patients aged ≥ 65 years.

**Methods:**

Data were collected from an active register of 102 internal medicine and geriatric wards in Italy (RePoSi project). Patients were recruited during four index weeks of a year. Socio-demographic data, reason for hospitalization, diagnoses, treatment, severity and comorbidity indexes (Cumulative Illness rating Scale CIRS-SI and CIRS-CI), renal function, functional (Barthel Index), and cognitive status (Short Blessed Test) and mood disorders (Geriatric Depression Scale) were recorded. Mortality rates were assessed in hospital 3 and 12 months after discharge.

**Results:**

Of the 4714 elderly patients hospitalized, 361 had a glycemia level ≥ 250 mg/dL at admission. Compared to subjects with lower glycemia level, patients with glycemia ≥ 250 mg/dL showed higher rates of male sex, smoke and class III obesity. These patients had a significantly lower Barthel Index (*p* = 0.0249), higher CIRS-SI and CIRS-CI scores (*p* = 0.0025 and *p* = 0.0013, respectively), and took more drugs. In-hospital mortality rate was 9.2% and 5.1% in subjects with glycemia ≥ 250 and < 250 mg/dL, respectively (*p* = 0.0010). Regression analysis showed a strong association between in-hospital death and glycemia ≥ 250 mg/dL (OR 2.07; [95% CI 1.34–3.19]), Barthel Index ≤ 40 (3.28[2.44–4.42]),

CIRS-SI (1.87[1.27–2.77]), and male sex (1.54[1.16–2.03]).

**Conclusions:**

The stronger predictors of in-hospital mortality for older patients admitted in general wards were glycemia level ≥ 250 mg/dL, Barthel Index ≤ 40, CIRS-SI, and male sex.

## Introduction

The number of adults with diabetes is raised from 108 million of 1980 to 422 million of 2014, worldwide [1]. This dramatic rise is largely due to the development of the “Diabesity” epidemic (type 2 diabetes and obesity) that represents the largest global epidemic in human history [2]. Diabetes directly caused 1.5 million of death in 2012, and 2.2 million of deaths were imputable to hyperglycemia [1].

Hyperglycemia is found in approximately 38% of hospitalized patients admitted to general wards, and diabetes represented the fourth most common disease mentioned on hospital discharges [4]. The association between increased glucose values and adverse hospital outcomes in hospitalized subjects with and without diabetes are well known [3]. The same results are well defined, when a different marker for poor glycemic control, such as glycemic variability was considered. A significant association between high glycemic variability and longer length of hospital stay and increased 3-month mortality in non-critical ill patients has been found [5]. However, the beneficial effects of better glycemic control in patients hospitalized in internal medicine wards are not clearly established and contribute to the efforts to achieve a better glycemic control in this setting. According to Ena and colleagues, there is an important gap between the clinical guidelines and both the management and the grade of glycemic control of diabetic subjects hospitalized in Spanish internal medicine wards [6]. Contrasting data are available about association between hyperglycemia at admission and main outcomes in subjects hospitalized in internal medicine wards.

A recent observational retrospective study did not find any correlation between mean glucose level, glycemic variability or persistent hyperglycemia and in-hospital mortality in elderly patients with community acquired pneumonia or chronic obstructive pulmonary disease admitted to internal medicine services [7] as well as according to the RePoSi register disability and not diabetes was a strong predictor of mortality in hospitalized elderly patients with pneumonia [8]. On the contrary, an association between admission glucose levels and in-hospital mortality and long of hospital stay was found in patients admitted to tertiary care hospital [9].

For this reason, the aim of this study was to determine if the identification of a simple measure of elevated glucose level at the time of admission was associated with comorbidities and outcomes in a cohort of elderly hospitalized in Internal Medicine and Geriatric Wards participating to the RePoSI (REgistro per lo studio delle POlipatologie e politerapie SImi) register study.

### Materials and methods

We retrospectively analyzed the data collected in the frame of the RePoSI (Registro Politerapie SIMI) project. RePoSI is an independent and ongoing collaborative register, organized by the Mario Negri Institute for Pharmacological Research and the Italian Society of Internal Medicine (SIMI). It encompassed the setting-up of a network of 102 internal medicine and geriatric wards that collected information about polytherapy on elderly patients with several diseases. Patients' eligibility criteria were: (1) admission to one of the attendee’s wards during the four index weeks chosen for recruitment (one in February, one in June, one in September, and one in December); (2) age ≥ 65 years; (3) having given informed consent. At least ten consecutive eligible patients were enrolled during each index week, recording data on socio-demographic details, the main reason for admission and comorbidities, diagnoses, treatment (including all drugs taken at hospital admission and recommended at discharge), clinical events during hospitalization and outcome. All participating centers had to complete the registration of all patients admitted, indicating those who were consecutively enrolled. For patients who were excluded, the reason had to be given. Also, data on mortality or any new hospitalization were collected, with a telephone interview to the patient or his/her relatives, 3 and 12 months after hospital discharge. Then, a final database was created and checked by the Mario Negri Institute for Pharmacological Research. The RePoSI study was approved by the Ethics Committee of each participating centre. All the details of database and population characteristics may be found in previous publication [10]. The project’s design is accessible at the related website (https://reposi.org/).

The dataset relating to 4714 patients was used for all the analyses. Socio-demographic variables such as age class, marital status, living arrangement and hospital admissions were all considered. As clinical characteristics, we evaluated: disease distribution at hospital admission (classification was based on the International Classification of Diseases-Ninth Revision), cognitive status tested with the Short Blessed Test (SBT) [11], mood disorders assessed by the Geriatric Depression Scale (GDS) [12], functional status at hospital admission (measured by means of the Barthel Index) [13], kidney function by means of eGFR (calculated using the Chronic Kidney Disease Epidemiology Collaboration formula) [14], severity and comorbidity indexes (evaluated, respectively, by the Cumulative Illness rating Scale CIRS-SI and CIRS-CI) [15] and in-hospital, 3-month and 1-year mortality rates.

### Statistical analysis

Data were reported as percentages for categorical variables and as means (95% confidence intervals) for quantitative variables. A Barthel Index score of ≤40 was used to select patients with significant disability according to our population characteristic. According to ROC curve analysis, a 250 mg/dl glycemic threshold was chosen as the best cut-off value for predicting mortality in our dataset. The comparison between groups was made using Fisher's exact-test for contingency tables and the z-test for comparison of proportions. The non-parametric Mann–Whitney-U-test was used for comparison of quantitative variables. A multivariate logistic analysis was used to explore the relationship between variables and outcomes (in-hospital and 3-month follow-up mortality). Odds ratios and 95% confidence-intervals were computed. Variables were chosen according to the Hosmer–Lemeshow methodology [16]: after univariate analysis, only variables with a *p*<0.20 were included in the final model; then, through a backward process, variables were excluded until a significance level of *p*<0.20 was reached for each variable. A two-tailed *p*<0.05 was considered statistically significant. Stata Statistical Software 2016, Release14 (Stata-Corp, College-Station, TX-USA) was used for database management and all the analyses.

## Results

This analysis included 4714 elderly inpatients admitted to the internal and geriatric wards of RePoSi project. Among 361 patients with a glycemia level ≥250 mg/dL, 54.8% were male and the mean age was 79.3 years (78.5–80.0), 7.3% were previously institutionalized and more than one third of patients were previously hospitalized. More than half of patients have a caregiver and were smokers or ex-smokers, and 3.4% were subjects with class III obesity (Table 1).

**Table 1:** Socio-demographic characteristics and some modifiable risk factors of the REPOSI elderly population according to fasting glucose ≥ 250 mg/dL categorization

Variables	Inpatient with Fasting glucose > 250 mg/dL	Inpatient with Fasting glucose ≤ 250 mg/dL	*p*
N° of subjects	361	4353	/
Men (%)	54.8	48.6	0.0222
Age*	79.3 (78.5—80.0)	79.5 (79.3—79.7)	0.5866
**Marital status (%)**			0.806
*Married*	54.0	53.9	
*Widow*	35.1	36.9	
*Separated*	1.1	1.3	
*Divorced*	1.4	1.3	
**Living arrangement (%)**			
*Alone*	20.1	23.0	0.016
*Spouse*	43.5	45.1	
*Sons*	15.7	15.0	
*Spouse and sons*	6.7	8.9	
*Other*	8.5	10.0	
Previously Institutionalized (%)	7.3	5.5	0.1633
Previously Hospitalized (%)	34.0	37.0	0.4157
**Caregiver (%)**	54.8	52.3	0.3749
*Spouse (%)*	33.5	33.2	0.646
*Brother/Sister (%)*	4.7	3.1	
*Son/Daughter (%)*	44.5	46.9	
*Son/Daughter in law (%)*	2.0	1.2	
*Grandson (%)*	2.6	3.8	
*Other (%)*	12.5	11.5	
Never Smoked (%)	47.9	55.0	0.038
ex-Smoker (%)	42.4	36.3	
Smoker (%)	9.5	8.5	
Never Alcohol (%)	55.2	56.7	0.189
Alcohol (%)	26.4	28.7	
ex-Alcohol (%)	8.4	5.8	
Casual Drinking (%)	9.8	8.6	
BMI*	26.48 (25.84—27.11)	25.85 (25.69—26.01)	0.1813
Underweight patients (%)^†^	3.8	4.0	0.8412
Optimal weight patients (%)^†^	40.7	41.1	0.9015
Overweight patients (%)^†^	34.8	35.3	0.8448
Class I obesity (%)^†^	11.0	12.9	0.3220
Class II obesity (%)^†^	4.7	3.0	0.1024
Class III obesity (%)^†^	3.4	1.4	0.0035

^*^ Data are reported as mean (95% Confidence Interval). ^†^Underweight – BMI (Body Mass Index) < 18.5 kg/m^2^, Optimal weight 18.5 to 24.9 kg/m^2^; overweight – BMI ≥ 25 to 29.9 kg/m^2^; class I obesity – BMI 30.0–34.9 kg/m^2^; class-II obesity – BMI: 35.0–39.9 kg/m^2^; class-III obesity – BMI ≥ 40.0 kg/m^2^)

**Fig. 1:**
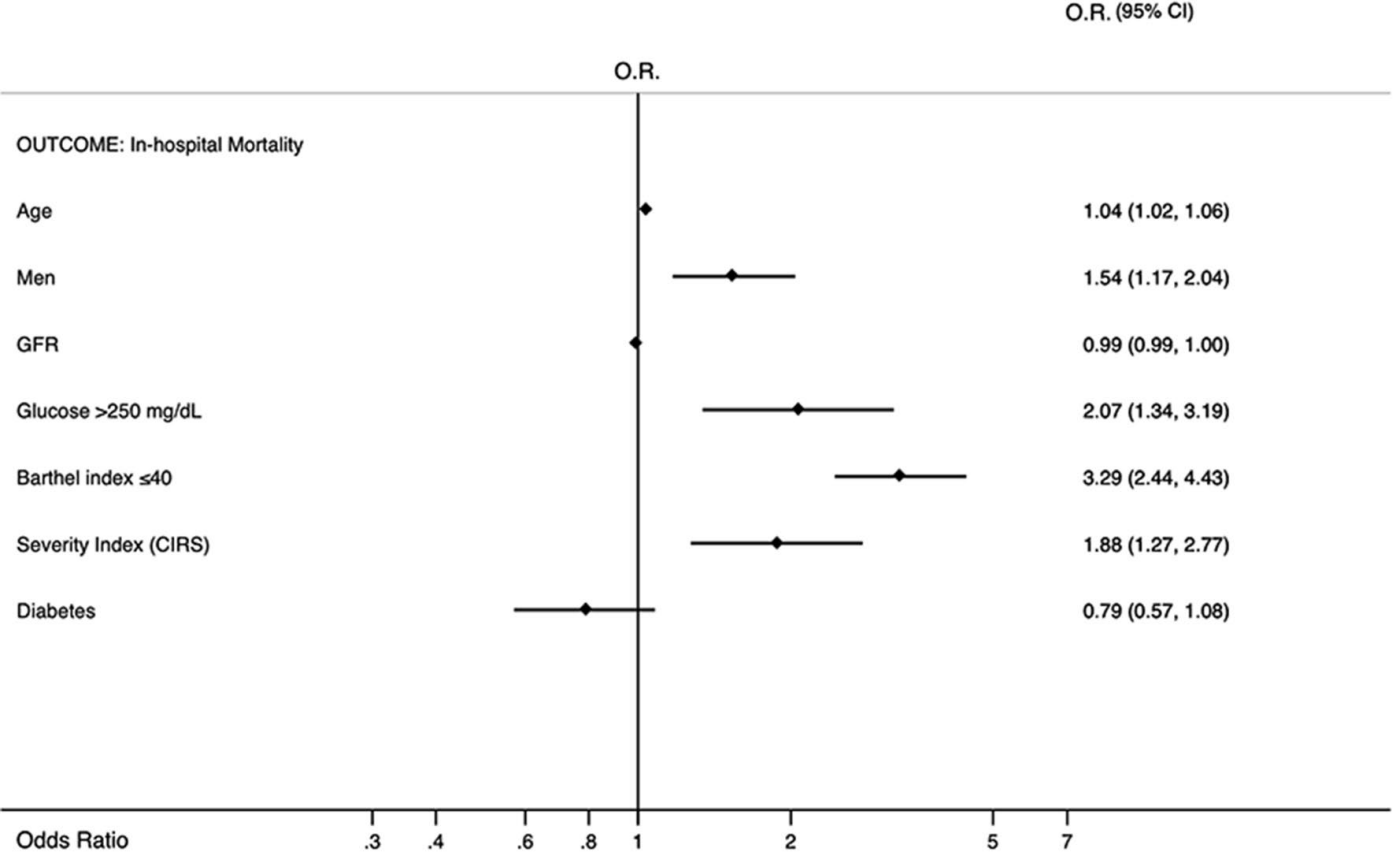
Multivariate Analysis in-hospital mortality OR = odds ratio; 95% CI = 95% confidence interval; GFR = Glomerular Filtration Rate calculated by CKD-EPI formula; CIRS = Cumulative Illness Rating Scale

Laboratory and clinical characteristics of inpatients with glycemia level at admission ≥250 mg/dL and <250 mg/dL are shown in Table 2. Subjects with glycemia level ≥250 mg/dL had higher count of leucocytes (*p*<0.0001) and platelets (*p*=0.0310), a lower glomerular filtration rate (*p*<0.0001), and 15.3% of them had a severe low glomerular filtration rate (*p*=0.0017), than those with blood glucose <250 mg/dL. Moreover, inpatients with glycemia level ≥250 mg/dL had a lower Barthel Index (*p*=0.0249), 18.8% had a Barthel Index ≤40 (*p*=0.0208), and 30.8% need for urinary catheter (*p*<0.0001). Compared to subjects with blood glucose <250 mg/dL, the group of patients with glycemia level ≥250 mg/dL had a significantly high CIRS for the evaluation of both severity and comorbidity indexes (*p*= 0.0025 and *p*= 0.0013, respectively) and they took more drugs at hospital admission (*p*=0.0020), at hospital discharge (*p*<0.0001), and at 3-month (*p*<0.0001) and 1-year follow-up (*p*=0.0099). Overall, disease distribution showed that diabetes, hypertension, chronic renal failure, ischemic heart disease, heart failure, anemia, atrial fibrillation, chronic obstructive pulmonary disease (COPD), cancer and dementia were more frequent in patients with glycemia ≥ 250 mg/dl (Table 3). The in-hospital mortality rate of patients with glycemia ≥250 mg/dL was 9.2% (*p*=0.0010) (Table 4). At 3-month discharge, 8.1% of patients with glycemia ≥250 mg/dL were institutionalized (*p*=0.0030). Glycemia ≥250 mg/dL (OR 2.07, 95% CI 1.34–3.19), Barthel Index ≤ 40 (OR 3.28, 95% CI 2.44–4.42), CIRS-SI (OR 1.87, 95% CI 1.27–2.77), and male sex (OR 1.54, 95% CI 1.16–2.03) were strong predictors of mortality at in-hospital mortality (Figure 1) while diabetes mellitus and glomerular filtration rate were protective. Moreover, age was an independent prognostic factor for in-hospital mortality. It is worth emphasizing that in the final model we verified that an interaction between rapid and long acting insulin was not present, and we did not report neither rapid and long-acting insulin neither anti-diabetic drugs because they were not statistically significant.

**Table 2:** Laboratory and clinical characteristics of the REPOSI population at hospital admission according to fasting glucose ≥ 250 mg/dL categorization

Variables	Inpatient with Fasting glucose ≥ 250 mg/dL	Inpatient with Fasting glucose < 250 mg/dL	*p*
Systolic blood pressure (mm Hg)*	131.1 (128.9—133.3)	131.9 (131.2—132.5)	0.5243
Diastolic blood pressure (mm Hg)*	73.1 (71.9—74.4)	73.5 (73.1—73.8)	0.6331
Heart rate (bpm)*	78.6 (76.6—80.5)	79.0 (78.5—79.5)	0.6200
Body temperature (°C)*	36.86 (36.66—37.06)	37.84 (36.07—39.60)	0.3157
Creatinine (mg/dL)*	1.43 (1.32—1.53)	1.25 (1.22—1.28)	< 0,0001
GFR*	53.0 (50.4—55.6)	59.7 (59.0—60.4)	< 0,0001
Mild decrease in GFR	33.1	41.1	0.0055
Moderate decrease in GFR	40.8	35.8	0.0800
Severe decrease in GFR	15.3	9.8	0.0017
Kidney Failure	4.1	3.5	0.5424
Hemoglobin (mg/dL)*	11.79 (11.54—12.05)	11.82 (11.75—11.89)	0.7198
Leucocytes (cells per microliter) (× 10^3^/μL) *	13.51 (9.00—18.02)	9.58 (9.14—10.03)	< 0,0001
Platelets (cells per microliter) (× 10^3^/μL) *	244.58 (230.42—258.75)	229.47 (226.19—232.74)	0.0310
Cholesterol (mg/dL)*	156.4 (149.5—163.3)	159.8 (158.2—161.3)	0.4724
Short Blessed Test score *	9.3 (8.4—10.2)	9.1 (8.8—9.3)	0.4924
Overt Cognitive impairment (Short Blessed Test score ≥ 10) (%)	43.1	44.0	0.7615
Need for urinary catheter (%)	30.8	21.8	0.0001
Barthel index score*	73.6 (70.2—77.1)	77.8 (76.9—78.7)	0.0249
Clinically significant disability			
(Barthel index ≤ 40) (%)	18.8	14.2	0.0208
Geriatric Depression Scale score*	1.47 (1.32—1.62)	1.38 (1.34—1.42)	0.2236
Probable Depression			
(Geriatric Depression Scale score > 2) (%)	19.5	18.3	0.6330
N° of drugs at hospital admission*	6.3 (6.0—6.7)	5.7 (5.6—5.8)	0.0020
N of in-hospital drugs*	8.6 (8.0—9.2)	7.8 (7.7—8.0)	0.0411
N of drugs at hospital discharge*	8.7 (8.2—9.2)	7.6 (7.5—7.7)	< 0.0001
N of drugs at follow-up 3 months*	7.6 (7.1—8.1)	6.5 (6.4—6.6)	< 0.0001
N of drugs at follow-up 1 year*	7.9 (6.7—9.1)	6.4 (6.1—6.7)	0.0099
Severity index (by CIRS) *	1.72 (1.68—1.76)	1.66 (1.65—1.67)	0.0025
Comorbidity index (by CIRS) *	3.38 (3.17—3.59)	3.02 (2.96—3.07)	0.0013

^*^ Data are reported as mean (95% Confidence Interval) BMI = Body Mass Index; CIRS = Cumulative Illness Rating Scale

**Table 3:** The most frequent clinical diagnoses (as percentage) in the REPOSI population according to fasting glucose ≥ 250 mg/dL categorization (the table only shows the diagnoses which frequency was more than 10%)

Comorbidities	Inpatient with Fasting glucose ≥ 250 mg/dL	Inpatient with Fasting glucose < 250 mg/dL	*p*
Diabetes	56.8	27.0	< 0.0001
Hypertension	54.3	59.5	0.0526
Chronic renal failure	27.4	19.6	0.0004
Ischemic heart disease	26.6	22.5	0.0766
Heart Failure	23.4	19.8	0.0984
Anemia	22.8	20.3	0.2638
Atrial fibrillation	21.0	24.9	0.0998
COPD	19.7	19.9	0.9280
Cancer	16.7	19.1	0.2966
Dementia	15.2	14.9	0.8769

**Table 4:** Length of hospital stay, destination at hospital discharge, in-hospital and at follow-up mortality of the whole REPOSI population according to fasting glucose ≥ 250 mg/dL categorization

Variables	Inpatient with fasting glucose ≥ 250 mg/dL	Inpatient with fasting glucose < 250 mg/dL	p
Length of hospital stay* (days)	11.4 (10.4—12.3)	11.8 (11.4—12.3)	0.7463
In-hospital mortality (%)	9.2	5.1	0.0010
3-month mortality (%)	7.0	9.6	0.2254
12-month mortality (%)	13.5	14.0	0.9300
*Destination at discharge (3-month)*			
*Home (%)*	84.8	89.6	0.0413
*Home care (%)*	3.3	3.2	0.9659
*Institution (%)*	8.1	3.7	0.0030
*Rehospitalization (%)*	3.8	3.5	0.8237
*Destination at discharge (12-month)*			
*Home (%)*	96.9	88.5	0.1421
*Home care (%)*	0.0	2.9	0.3310
*Institution (%)*	3.1	5.7	0.5310
*Rehospitalization (%)*	15.8	15.0	0.8951

^*^ Data are reported as means (95% Confidence Interval)

## Discussion

This study has investigated the possible association between elevated glucose levels at the time of admission and comorbidities along with short- and long-term mortality in hospitalized elderly people. Patients admitted to internal medicine wards have some risk for mortality independently of admitting diagnosis. This study highlighted the crucial role of glycemia upon admission ≥250 mg/dL that significantly increases the risk of death regarding in-hospital mortality in elderly subjects with and without a prior diagnosis of diabetes mellitus. Our results are in line with previous analysis that found an increased in-hospital mortality in subjects with the admission glucose level between 100 and 200 mg/dL [17]. On the other hand, in patients brought to the emergency room a plasma glucose level greater than 162 mg/dL (9 mmol/L) was a predictor of in-hospital mortality [18]. A very recent analysis of emergency visits from a Swedish hospital showed that in-hospital mortality was significantly higher only for subjects with severe hyperglycemia and significant higher percentage of 30-day mortality (4.0–4.5%) occurred in modest (>126 mg/dL ≤200 mg/dL, >7 ≤11.1 mmol/L) and severe hyperglycemia (≥200 mg/dL, ≥11.1 mmol/L) irrespective of diagnosis and treating medical specialty [19]. Our findings are in agreement with previous studies that found a strong association between severe hyperglycemia (glucose >200 mg/dL) and 30-day risk of mortality in critical ill patients with sepsis [20]. In this regard, some evidence showed that subjects with newly diagnosed hyperglycemia were more severe ill than patients with known diabetes or normoglycemic [3]; hyperglycemia represents an increase in blood glucose levels in response to physical stress and could indicate a more severe condition [21, 22]. On the other side, hyperglycemia could produce a more severe disease instead of being a marker of disease. In support of this hypothesis, intensive insulin therapy in critical ill patients results in a reduction in mortality and morbidity [23].

The importance of hyperglycemia could depend on the underlying medical condition and the level of stress. A slight hyperglycemia could represent an essential response producing beneficial effects [24]. Prolonged high levels of glucose could represent a maladaptive response determining the increase of reactive oxygen species and consequently cellular injury, intracellular and extracellular dehydration, electrolyte abnormalities, and depressing immune function [25].

In our patients, there was no association between diabetes and mortality. This finding is in agreement with previous studies which showed that critical ill patients with diabetes did not have an increased mortality and conversely had a decreased mortality [26]. Moreover, diabetes was not a strong predictor of mortality in hospitalized elderly patients with pneumonia [8]. A possible explanation could be due to the long-term effect of antidiabetic treatment or alternatively to a more appropriately attention of clinicians to the hyperglycemic state of admitted patients. Another important finding of our analysis concerns the fundamental role of Barthel index and CIRS. Recent studies showed that hyperglycemia was an independent factor of functional outcomes of patients with acute ischemic stroke as measured with Barthel index [27]. Barthel Index was a strong predictor of mortality at in-hospital, 3-month, and 1-year mortality. Functional disability represents a common risk factor in subjects aged ≥75 years [28]. The role of functional disability was found to be relevant to explain the risk of early readmissions in a cohort of patients aged 75 and older [29]. In addition, limited activities in daily living on hospital admission as measured by Barthel Index score less than forty-five were predictive of prolonged hospital stay independent of diagnosis. Whereas a Barthel Index less than sixty-five on admission predicts mortality within six months of discharge. Finally, functional disability on admission was predictive of institutionalization on discharge [30]. Higuchi et al. showed that the Barthel Index as indicator of activities of daily living may be a useful predictor for 1-year mortality in very elderly patients undergoing percutaneous coronary intervention for acute coronary syndrome [31].

Regarding the important role of CIRS-SI, our findings are in line with a recent analysis that showed that CIRS assessment of comorbidity burden is the more useful clinical tool for the evaluation of length of hospital stay and all-cause mortality in hospitalized elderly patients [32]. In addition, CIRS represents the instrument of choice for multimorbidity assessment in clinical trials and has the benefit to predict mortality hospital readmission and prolonged hospital stay [33, 34]. CIRS ≥ 3 at discharge was significantly associated with a risk of hospital readmission within 3 months [35].

CIRS-SI was a strong predictor of mortality at in-hospital and 1-year mortality. CIRS-SI and CIRS CI were higher in patients affected by pneumonia in comparison with people without pneumonia [8]. It is worth mentioning that a high index of comorbidity (CIRS index >3) is significantly associated with gastrointestinal bleeding in elderly patients [36].

Another important finding was the role of male sex. Our data are consistent with previous analysis that showed that male sex was more affected by cumulative illness burden [10, 37]. Moreover, male sex was associated with increased number of cardio-renal-metabolic diseases among patients with type 2 diabetes [38].

The major strength of the study is the multicentre design of the REPOSI register and the large number of internal medicine and geriatric wards involved that make the study representative of the real-world scenario. A major limitation of this study concerns the lack of clinical information, particularly nutritional status, that is beyond the purpose of the RePoSI study. Moreover, we did not collect data that could influence outcome such as glycemia levels measurements during hospitalization and hypoglycemic events.

## Conclusions

In conclusion, our analysis revealed that a single blood glucose level taken at the time of admission was associated with comorbidities and short-term outcomes in the real-world scenario of a cohort of elderly hospitalized in Internal Medicine and Geriatric Wards. Further studies are necessary to evaluate whether intervention and normalization of blood glucose levels in these individuals make a difference to short- and long-term outcomes.

## Data Availability

The datasets generated during and/or analyzed during the current study are available from the corresponding author on reasonable request.

## Appendix: Investigators and co-authors of the REPOSI (REgistro POliterapie SIMI, Società Italiana di Medicina Interna) Study Group are as follows

### Steering Committee

Pier Mannuccio Mannucci *(Chair) (Fondazione IRCCS Cà Granda Ospedale Maggiore Policlinico, Milano)*, Alessandro Nobili *(co-chair) (Istituto di Ricerche Farmacologiche Mario Negri IRCCS, Milano),* Antonello Pietrangelo *(Presidente SIMI),* Francesco Perticone *(Direttore CRIS – SIMI),* Giuseppe Licata *(Socio d’onore SIMI),* Francesco Violi *(Policlinico Umberto I, Roma, Prima Clinica Medica),* Gino Roberto Corazza, *(Reparto 11, IRCCS Policlinico San Matteo di Pavia, Pavia, Clinica Medica I),* Salvatore Corrao *(ARNAS Civico, Di Cristina, Benfratelli, DiBiMIS, Università di Palermo, Palermo),* Alessandra Marengoni *(Spedali Civili di Brescia, Brescia),* Francesco Salerno *(IRCCS Policlinico San Donato Milanese, Milano),* Matteo Cesari *(UO Geriatria, Università degli Studi di Milano),* Mauro Tettamanti, Luca Pasina, Carlotta Franchi *(Istituto di Ricerche Farmacologiche Mario Negri IRCCS, Milano).*

### Clinical data monitoring and revision

Carlotta Franchi, Laura Cortesi, Mauro Tettamanti, Gabriella Miglio *(Istituto di Ricerche Farmacologiche Mario Negri IRCCS, Milano).*

### Database Management and Statistics

Mauro Tettamanti, Laura Cortesi, Ilaria Ardoino, Alessio Novella *(Istituto di Ricerche Farmacologiche Mario Negri IRCCS, Milano).*

## Investigators

Domenico Prisco, Elena Silvestri, Giacomo Emmi, Alessandra Bettiol, Cenci Caterina *(Azienda Ospedaliero Universitaria Careggi Firenze, Medicina Interna Interdisciplinare);*

Gianni Biolo, Michela Zanetti, Martina Guadagni, Michele Zaccari, Massimiliano Chiuch, Michele Zaccari (*Azienda Sanitaria Universitaria Integrata di Trieste, Clinica Medica Generale e Terapia Medica*);

Massimo Vanoli, Giulia Grignani, Edoardo Alessandro Pulixi *(Azienda Ospedaliera della Provincia di Lecco, Ospedale di Merate, Lecco, Medicina Interna);*

Mauro Bernardi, Silvia Li Bassi, Luca Santi, Giacomo Zaccherini *(Azienda Ospedaliera Policlinico Sant’Orsola-Malpighi, Bologna, Semeiotica Medica Bernardi);*

Graziana Lupattelli, Elmo Mannarino, Vanessa Bianconi, Francesco Paciullo, Riccardo Alcidi *(Azienda Ospedaliera Santa Maria della Misericordia, Perugia, Medicina Interna);*

Ranuccio Nuti, Roberto Valenti, Martina Ruvio, Silvia Cappelli, Alberto Palazzuoli *(Azienda Ospedaliera Università Senese, Siena, Medicina Interna I);*

Domenico Girelli, Fabiana Busti, Giacomo Marchi *(Azienda Ospedaliera Universitaria Integrata di Verona, Verona, Medicina Generale e Malattie Aterotrombotiche e Degenerative);*

Mario Barbagallo, Ligia Dominguez, Floriana Cocita, Vincenza Beneduce, Lidia Plances *(Azienda Ospedaliera Universitaria Policlinico Giaccone Policlinico di Palermo, Palermo, Unità Operativa di Geriatria e Lungodegenza);*

Salvatore Corrao, Giuseppe Natoli, Salvatore Mularo, Massimo Raspanti, Christiano Argano *(A.R.N.A.S. Civico, Di Cristina, Benfratelli, Palermo, UOC Medicina Interna ad Indirizzo Geriatrico-Riabilitativo);*

Marco Zoli, Ilaria Lazzari, Mattia Brunori, Elisa Fabbri, Donatella Magalotti, Raffaella Arnò *(Azienda Ospedaliera Universitaria Policlinico S. Orsola-Malpighi, Bologna, Unità Operativa di Medicina Interna);*

Franco Laghi Pasini, Pier Leopoldo Capecchi, *(Azienda Ospedaliera Universitaria Senese, Siena, Unità Operativa Complessa Medicina 2);*

Giuseppe Palasciano, Maria Ester Modeo, Carla Di Gennaro *(Azienda Ospedaliero-Universitaria Consorziale Policlinico di Bari, Bari, Medicina Interna Ospedaliera "L. D'Agostino", Medicina Interna Universitaria “A. Murri”);*

Maria Domenica Cappellini, Diletta Maira, Valeria Di Stefano, Giovanna Fabio, Sonia Seghezzi, Marta Mancarella, Margherita Migone De Amicis, Giacomo De Luca, Natalia Scaramellini *(Fondazione IRCCS Cà Granda Ospedale Maggiore Policlinico, Milano, Unità Operativa Medicina Interna IA);*

Matteo Cesari, Paolo Dionigi Rossi, Sarah Damanti, Marta Clerici, Federica Conti, Giulia Bonini, Barbara Brignolo Ottolini *(Fondazione IRCCS Cà Granda Ospedale Maggiore Policlinico, Milano, Geriatria);*

Antonio Di Sabatino, Emanuela Miceli, Marco Vincenzo Lenti, Martina Pisati, Costanza Caccia Dominioni *(IRCCS Policlinico San Matteo di Pavia, Pavia, Clinica Medica I, Reparto 11);*

Giovanni Murialdo, Alessio Marra, Federico Cattaneo, Roberto Pontremoli, Valentina Beccati, Giulia Nobili *(IRCCS Azienda Ospedaliera Universitaria San Martino-IST di Genova, Genova, Clinica di Medicina Interna 2);*

Maria Beatrice Secchi, Davide Ghelfi *(Ospedale Bassini di Cinisello Balsamo, Milano, Divisione Medicina);*

Luigi Anastasio, Lucia Sofia, Maria Carbone *(Ospedale Civile Jazzolino di Vibo Valentia, Vibo Valentia, Medicina interna);*

Francesco Cipollone, Maria Teresa Guagnano, Emanuele Valeriani, Ilaria Rossi *(Ospedale Clinicizzato SS. Annunziata, Chieti, Clinica Medica);*

Gerardo Mancuso, Daniela Calipari, Mosè Bartone *(Ospedale Giovanni Paolo II Lamezia Terme, Catanzaro, Unità Operativa Complessa Medicina Interna);*

Giuseppe Delitala, Maria Berria, Chiara Pes, Alessandro Delitala *(Azienda ospedaliera-universitaria di Sassari, Clinica Medica);*

Maurizio Muscaritoli, Alessio Molfino, Enrico Petrillo *(Policlinico Umberto I, Sapienza Università di Roma, Medicina Interna e Nutrizione Clinica Policlinico Umberto I);*

Giuseppe Zuccalà, Gabriella D’Aurizio *(Policlinico Universitario A. Gemelli, Roma, Roma, Unità Operativa Complessa Medicina d'Urgenza e Pronto Soccorso).*

Giuseppe Romanelli, Alessandra Marengoni, Alberto Zucchelli, Francesca Manzoni, Andrea Volpini *(Spedali Civili di Brescia, Brescia, Geriatria);*

Antonio Picardi, Umberto Vespasiani Gentilucci, Paolo Gallo, Chiara Dell’Unto *(Università Campus Bio-Medico, Roma, Medicina Clinica-Epatologia);*

Giorgio Annoni, Maurizio Corsi, Giuseppe Bellelli, Sara Zazzetta, Paolo Mazzola, Hajnalka Szabo, Alessandra Bonfanti *(Università degli studi di Milano-Bicocca Ospedale S. Gerardo, Monza, Unità Operativa di Geriatria);*

Franco Arturi, Elena Succurro, Mariangela Rubino, Bruno Tassone, Giorgio Sesti *(Università degli Studi Magna Grecia, Policlinico Mater Domini, Catanzaro, Unità Operativa Complessa di Medicina Interna);*

Maria Grazia Serra, Maria Antonietta Bleve *(Azienda Ospedaliera "Cardinale Panico" Tricase, Lecce, Unità Operativa Complessa Medicina);*

Laura Gasbarrone, Maria Rosaria Sajeva *(Azienda Ospedaliera Ospedale San Camillo Forlanini, Roma, Medicina Interna 1);*

Antonio Brucato, Silvia Ghidoni *(Azienda Ospedaliera Papa Giovanni XXIII, Bergamo, Medicina 1);*

Fabrizio Fabris, Irene Bertozzi, Giulia Bogoni, Maria Victoria Rabuini, Elisabetta Cosi, Paolo Scarinzi, Annalisa Amabile, Elisabetta Omenetto, Tancredi Prandini *(Azienda Ospedaliera Università di Padova, Padova, Clinica Medica I);*

Roberto Manfredini, Fabio Fabbian, Benedetta Boari, Alfredo De Giorgi, Ruana Tiseo, Roberto De Giorgio *(Azienda Ospedaliera—Universitaria Sant'Anna, Ferrara, Unità Operativa Clinica Medica);*

Giuseppe Paolisso, Maria Rosaria Rizzo *(Azienda Ospedaliera Universitaria della Seconda Università degli Studi di Napoli, Napoli, VI Divisione di Medicina Interna e Malattie Nutrizionali dell'Invecchiamento);*

Claudio Borghi, Enrico Strocchi, Eugenia Ianniello, Mario Soldati *(Azienda Ospedaliera Universitaria Policlinico S. Orsola-Malpighi, Bologna, Unità Operativa di Medicina Interna Borghi);*

Carlo Sabbà, Francesco Saverio Vella, Patrizia Suppressa, Andrea Schilardi, Francesca Loparco, Giovanni Michele De Vincenzo, Alessio Comitangelo, Emanuele Amoruso *(Azienda Ospedaliero-Universitaria Consorziale Policlinico di Bari, Bari, Medicina Interna Universitaria C. Frugoni);*

Luigi Fenoglio, Andrea Falcetta, Christian Bracco *(Azienda Sanitaria Ospedaliera Santa Croce e Carle di Cuneo, Cuneo, S. C. Medicina Interna);*

Anna L. Fracanzani Silvia Fargion,, Silvia Tiraboschi, Annalisa Cespiati, Giovanna Oberti, Giordano Sigon *(Fondazione IRCCS Cà Granda Ospedale Maggiore Policlinico, Milano, Medicina Interna 1B);*

Flora Peyvandi, Raffaella Rossio, Barbara Ferrari, Giulia Colombo, Pasquale Agosti *(Fondazione IRCCS Cà Granda Ospedale Maggiore Policlinico, Milano, UOC Medicina generale – Emostasi e trombosi);*

Valter Monzani, Valeria Savojardo, Christian Folli, Giuliana Ceriani *(Fondazione IRCCS Cà Granda Ospedale Maggiore Policlinico, Milano, Medicina Interna Alta Intensità);*

Francesco Salerno, Giada Pallini *(IRCCS Policlinico San Donato e Università di Milano, San Donato Milanese, Medicina Interna);*

Franco Dallegri, Luciano Ottonello, Luca Liberale, Lara Caserza, Kassem Salam *(Università di Genova, Genova, Medicina Interna 1);*

Nicola Lucio Liberato, Tiziana Tognin (*ASST di Pavia, UOSD Medicina Interna, Ospedale di Casorate Primo, Pavia);*

Giovanni Battista Bianchi, Sabrina Giaquinto (*Ospedale "SS Gerosa e Capitanio" di Lovere, Bergamo, Unità Operativa Complessa di Medicina Generale, Azienda Ospedaliera "Bolognini" di Seriate, Bergamo);*

Francesco Purrello, Antonino Di Pino, Salvatore Piro *(Ospedale Garibaldi Nesima, Catania, Unità Operativa Complessa di Medicina Interna);*

Renzo Rozzini, Lina Falanga, Elena Spazzini, Camillo Ferrandina *(Ospedale Poliambulanza, Brescia, Medicina Interna e Geriatria);*

Giuseppe Montrucchio, Paolo Petitti, Paolo Peasso, Edoardo Favale, Cesare Poletto *(Dipartimento di Scienze Mediche, Università di Torino, Città della Scienza e della Salute, Torino, Medicina Interna 2 U. Indirizzo d'Urgenza);*

Raffaella Salmi, Piergiorgio Gaudenzi *(Azienda Ospedaliera-Universitaria S. Anna, Ferrara, Unità Operativa di Medicina Ospedaliera II);*

Francesco Violi, Ludovica Perri *(Policlinico Umberto I, Roma, Prima Clinica Medica);*

Raffaele Landolfi, Massimo Montalto, Antonio Mirijello *(Policlinico Universitario A. Gemelli, Roma, Clinica Medica);*

Luigina Guasti, Luana Castiglioni, Andrea Maresca, Alessandro Squizzato, Leonardo Campiotti, Alessandra Grossi *(Università degli Studi dell'Insubria, Ospedale di Circolo e Fondazione Macchi, Varese, Medicina Interna I);*

Marco Bertolotti, Chiara Mussi, Giulia Lancellotti, Maria Vittoria Libbra, Giulia Dondi, Elisa Pellegrini, Lucia Carulli, Matteo Galassi, Yasmine Grassi *(Università di Modena e Reggio Emilia, Azienda Ospedaliero-Universitaria di Modena; Ospedale Civile di Baggiovara, Unità Operativa di Geriatria);*

Francesco Perticone, Maria Perticone, Rosa Battaglia, Marco FIlice, Raffaele Maio *(Università Magna Grecia Policlinico Mater Domini, Catanzaro, Unità Operativa Malattie Cardiovascolari Geriatriche);*

Vincenzo Stanghellini, Eugenio Ruggeri, Sara del Vecchio *(Dipartimento di Scienze Mediche e Chirurgiche, Unità Operativa di Medicina Interna, Università degli Studi di Bologna/Azienda Ospedaliero-Universitaria S.Orsola-Malpighi, Bologna);*

Andrea Salvi, Roberto Leonardi, Giampaolo Damiani *(Spedali Civili di Brescia, U.O. 3a Medicina Generale);*

William Capeci, Armando Gabrielli, Massimo Mattioli, Giuseppe Pio Martino, Lorenzo Biondi, Pietro Pettinari *(Clinica Medica, Azienda Ospedaliera Universitaria—Ospedali Riuniti di Ancona);*

Riccardo Ghio, Anna Dal Col *(Azienda Ospedaliera Università San Martino, Genova, Medicina III);*

Salvatore Minisola, Luciano Colangelo, Mirella Cilli, Giancarlo Labbadia *(Policlinico Umberto I, Roma, SMSC03—Medicina Interna A e Malattie Metaboliche dell'osso);*

Antonella Afeltra, Benedetta Marigliano, Maria Elena Pipita *(Policlinico Campus Biomedico Roma, Roma, Medicina Clinica);*

Pietro Castellino, Luca Zanoli, Samuele Pignataro, Alfio Gennaro, Julien Blanco (*Azienda Ospedaliera Universitaria Policlinico – V. Emanuele, Catania, Dipartimento di Medicina);*

Valter Saracco, Marisa Fogliati, Carlo Bussolino *(Ospedale Cardinal Massaia Asti, Medicina A);*

Francesca Mete, Miriam Gino *(Ospedale degli Infermi di Rivoli, Torino, Medicina Interna).*

Antonio Cittadini, Carlo Vigorito, Michele Arcopinto, Andrea Salzano, Emanuele Bobbio, Alberto Maria Marra, Domenico Sirico *(Azienda Policlinico Universitario Federico II di Napoli, Napoli, Medicina Interna e Riabilitazione Cardiologica);*

Guido Moreo, Francesca Gasparini, Silvia Prolo, Gloria Pina *(Clinica San Carlo Casa di Cura Polispecialistica, Paderno Dugnano, Milano, Unità Operativa di Medicina Interna);*

Alberto Ballestrero, Fabio Ferrando *(Clinica Di Medicina Interna ad Indirizzo Oncologico, Azienda Ospedaliera Università San Martino di Genova);*

Sergio Berra, Simonetta Dassi, Maria Cristina Nava *(Medicina Interna, Azienda Ospedaliera Guido Salvini, Garnagnate, Milano);*

Bruno Graziella, Stefano Baldassarre, Salvatore Fragapani, Gabriella Gruden *(Medicina Interna III, Ospedale S. Giovanni Battista Molinette, Torino);*

Giorgio Galanti, Gabriele Mascherini, Cristian Petri, Laura Stefani *(Agenzia di Medicina dello Sport, AOUC Careggi, Firenze);*

Margherita Girino, Valeria Piccinelli (Medicina Interna, Ospedale S. Spirito Casale Monferrato, Alessandria);

Francesco Nasso, Vincenza Gioffrè, Maria Pasquale *(Struttura Operativa Complessa di Medicina Interna, Ospedale Santa Maria degli Ungheresi, Reggio Calabria);*

Giuseppe Scattolin, Sergio Martinelli, Mauro Turrin *(Medicina Interna, Ospedale di Monselice, Padova);*

Leonardo Sechi, Cristina Catena, Gianluca Colussi *(Clinica Medica, Azienda Ospedaliera Universitaria, Udine).*

Nicola Passariello, Luca Rinaldi *(Presidio Medico di Marcianise, Napoli, Medicina Interna);*

Franco Berti, Giuseppe Famularo, Patrizia Tarsitani *(Azienda Ospedaliera San Camillo Forlanini, Roma, Medicina Interna II);*

Roberto Castello, Michela Pasino *(Ospedale Civile Maggiore Borgo Trento, Verona, Medicina Generale e Sezione di Decisione Clinica);*

Gian Paolo Ceda, Marcello Giuseppe Maggio, Simonetta Morganti, Andrea Artoni *(Azienda Ospedaliero Universitaria di Parma, U.O.C Clinica Geriatrica);*

Stefano Del Giacco, Davide Firinu, Francesca Losa, Giovanni Paoletti, Giulia Costanzo *(Policlinico Universitario Duilio Casula, Azienda Ospedaliero-Universitaria di Cagliari, Cagliari, Medicina Interna, Allergologia ed Immunologia Clinica);*

Giuseppe Montalto, Anna Licata, Valentina Malerba, Filippo Alessandro Montalto *(Azienda Ospedaliera Universitaria Policlinico Paolo Giaccone, Palermo, UOC di Medicina Interna);*

Antonino Lasco, Giorgio Basile, Antonino Catalano *(Azienda Ospedaliera Universitaria Policlinico G. Martino, Messina, Unità Operativa di Geriatria);*

Lorenzo Malatino, Benedetta Stancanelli, Valentina Terranova, Salvatore Di Marca, Rosario Di Quattro, Lara La Malfa, Rossella Caruso (Azienda Ospedaliera per l'Emergenza Cannizzaro, Catania, Clinica Medica Università di Catania);

Patrizia Mecocci, Carmelinda Ruggiero, Virginia Boccardi *(Università degli Studi di Perugia-Azienda Ospedaliera S.M. della Misericordia, Perugia, Struttura Complessa di Geriatria);*

Tiziana Meschi, Fulvio Lauretani, Andrea Ticinesi, Antonio Nouvenne *(Azienda Ospedaliera Universitaria di Parma, U.O Medicina Interna e Lungodegenza Critica);*

Pietro Minuz, Luigi Fondrieschi *(Azienda Ospedaliera Universitaria Verona, Policlinico GB Rossi, Verona, Medicina Generale per lo Studio ed il Trattamento dell’Ipertensione Arteriosa);*

Mario Pirisi, Gian Paolo Fra, Daniele Sola *(Azienda Ospedaliera Universitaria Maggiore della Carità, Medicina Interna 1);*

Massimo Porta,Piero Riva *(Azienda Ospedaliera Universitaria Città della Salute e della Scienza di Torino, Medicina Interna 1U);*

Roberto Quadri, Erica Larovere, Marco Novelli *(Ospedale di Ciriè, ASL TO4, Torino, S.C. Medicina Interna);*

Giorgio Scanzi, Caterina Mengoli, Stella Provini, Laura Ricevuti *(ASST Lodi, Presidio di Codogno, Milano, Medicina);*

Emilio Simeone, Rosa Scurti, Fabio Tolloso *(Ospedale Spirito Santo di Pescara, Geriatria);*

Roberto Tarquini, Alice Valoriani, Silvia Dolenti, Giulia Vannini *(Ospedale San Giuseppe, Empoli, USL Toscana Centro, Firenze, Medicina Interna I);*

Alberto Tedeschi, Lucia Trotta *(ASST Fatebenefratelli—Sacco, Milano, Medicina Interna a indirizzo Pneumologico);*

Riccardo Volpi, Pietro Bocchi, Alessandro Vignali *(Azienda Ospedaliera Universitaria di Parma, Clinica e Terapia Medica).*

Sergio Harari, Chiara Lonati, Mara Cattaneo, Federico Napoli (*Ospedale San Giuseppe Multimedica Spa, U.O. Medicina Generale*).
