# Correction to: Hyperglycemia at admission, comorbidities, and in-hospital mortality in elderly patients hospitalized in internal medicine wards: data from the RePoSI Registry

**DOI:** 10.1007/s00592-021-01749-z

**Published:** 2021-06-17

**Authors:** Salvatore Corrao, Alessandro Nobili, Giuseppe Natoli, Pier Mannuccio Mannucci, Francesco Perticone, Antonello Pietrangelo, Christiano Argano, the REPOSI Investigators

**Affiliations:** 1 Department of Internal Medicine, UOC Medicina Interna 2 iGR, National Relevance Hospital Trust, ARNAS Civico, Di Cristina e Benfratelli, Piazza Nicola Leotta, 4 – 90127 Palermo, Italy; 2 Biomedical Department of Internal Medicine and Medical Specialties (DiBiMIS), University of Palermo, Palermo, Italy; 3 Department of Neuroscience, IRCCS Istituto Di Ricerche Farmacologiche Mario Negri, Milan, Italy; 4 Scientific Direction, IRCCS Foundation Maggiore Hospital Policlinico, Milan, Italy; 5 Department of Medical and Surgical Sciences, University Magna Graecia of Catanzaro, Catanzaro, Italy; 6 Department of Internal Medicine II, CenterforHemochromatosis, University of Modena and Reggio Emilia Policlinico, Modena, Italy

## Correction to: Acta Diabetologica 10.1007/s00592-021-01716-8

Authors would like to update the list of Reposi Investigators as collaborators and update "on behalf of the RePoSi Collaborators" to the end of authors list.

The original article has been corrected.

